# Adherence as a Predictor of Sexual Behaviors in People Living with HIV/AIDS during the First Year of Antiretroviral Therapy in Rural Cameroon: Data from Stratall ANRS 12110/ESTHER Trial

**DOI:** 10.1371/journal.pone.0036118

**Published:** 2012-06-06

**Authors:** Gilbert Ndziessi, Sylvie Boyer, Charles Kouanfack, Julien Cohen, Fabienne Marcellin, Jean-Paul Moatti, Eric Delaporte, Bruno Spire, Christian Laurent, Maria Patrizia Carrieri, for the Stratall ANRS 12110/ESTHER Study Group

**Affiliations:** 1 INSERM, U912 (SESSTIM), Marseille, France; 2 Université Aix Marseille, IRD, UMR-S912, Marseille, France; 3 ORS PACA, Observatoire Régional de la Santé Provence Alpes Côte d’Azur, Marseille, France; 4 Central Hospital, Yaoundé, Cameroon; 5 Institut de Recherche pour le Développement (IRD), University Montpellier 1, UMI 233 Montpellier, France; 6 Department of Infectious and Tropical Diseases, University Hospital, Montpellier, France; National Institute of Allergy and Infectious Diseases, United States of America

## Abstract

**Objective:**

This study aims to investigate the time pattern of inconsistence condom use (ICU) during the first year of antiretroviral therapy (ART) and its relationship with treatment adherence in naïve HIV-infected adult patients.

**Methods:**

Data collection was nested within a longitudinal trial on HIV treatment. ICU was defined as reporting to have “never”, “sometimes” or “nearly always” used condoms with one’s main or casual partner(s) - either HIV-negative or of unknown HIV status in the three previous months. Adherence was defined as taking 100% of their ART prescribed doses in the 4 days before the visit and “not having interrupted treatment”, even once, for more than two consecutive days during the 4 previous weeks. Mixed logistic regression was used to study the relationship between adherence and ICU.

**Results:**

Among the 459 patients enrolled, 212 (46%) during 334 visits reported to have had sexual intercourse at least once with their partner(s) – either HIV-negative or of unknown HIV status- during the first 12 months of ART. The proportion of ICU was 76%, 50% and 59% at month 0 (M0), month 6 (M6) and month 12 (M12), while 60% and 66% of patients were ART-adherent at M6 and M12, respectively. After adjustment for the frequency of sexual activity, type of sexual partner(s), perceived social class and desire for a child, patients adherent to ART were less likely to report ICU when compared with baseline (AOR [95% CI]: 0.38 [0.19–0.76]; P = 0.006).

**Conclusions:**

Adherence to ART is associated with a lower risk of ICU but this result needs to be interpreted carefully. As adherence behaviors are not only determined by problems with the healthcare systems but also by social barriers encountered by patients in their daily life, counseling should not only be ART adherence-centered but also patient-centered, including sexual risk minimization and psychosocial support.

## Introduction

Since the introduction of the WHO “3 by 5” initiative [1], access to and use of health services providing antiretroviral therapy (ART) by people living with HIV/AIDS (PLWHA) in Sub-Saharan Africa has increased rapidly and substantially. However this progress in ART coverage remains concomitant with a high incidence of HIV infection, highlighting the need for substantially greater success in the prevention of new HIV infections [2]. While ART is increasingly regarded as a preventive intervention able to significantly reduce sexual transmission of HIV through decreased viremia [3], [4], sexual behaviors among PLWHA on ART remain a topic of major interest in Sub-Saharan Africa where the great majority of infections are attributable to sexual transmission [5]. On the one hand, several studies have confirmed that sustained adherence and continuity of ART - the basic conditions for treatment as prevention (TasP) [4] - remain a major challenge in Sub-Saharan countries where access to treatment is constrained by economic and structural barriers [6]. On the other hand, results of meta-analyses conducted both in high- and low-resource settings [7], [8] concluded that while ART-treated patients do not exhibit increased sexual risk behaviors, unprotected sex remains highly prevalent in Sub-Saharan African patients [8], [9], [10], including viremic patients, something also suggested by a study of Wandera et al. [10]. Few studies however have used longitudinal data to describe sexual behaviors during the course of ART or to assess the temporal relationship between psychosocial factors such as ART adherence and sexual behaviors. The relationship between ART adherence and unsafe sexual behaviors is of particular interest as the former may be considered a major component of the well-known “Swiss statement” which claims that there is a reduced likelihood of HIV sexual transmission in patients with a history of adherence and controlled viremia [4].

This analysis, based on longitudinal data of HIV-infected adults enrolled in the Stratall ANRS 12110/ESTHER trial in Cameroon, was designed to study the time pattern of unsafe sexual behaviors during the first 12 months of ART and its relationship with treatment adherence. However, as the study is focused on predictors of positive prevention (and not on unsafe sexual behaviors *per se*) we preferred to target individuals who had behaviors leading to a higher risk of HIV transmission– i.e., individuals with partners who were seronegative or whose serostatus was unknown.

## Materials and Methods

### Study Design

Longitudinal data collection, including psychosocial and sexual behavioral data, was nested within the randomized, 24-month open-label STRATALL ANRS 12110/ESTHER trial (ClinicalTrials.gov, NCT00301561) in Cameroon, designed to compare the effectiveness of clinical monitoring (n = 238 patients) versus clinical plus laboratory monitoring (viral load plus CD4) [11] (n = 221patients). The detailed methodology and main results of the STRATALL trial have already been reported elsewhere [12]. Briefly, 459 ART-naïve patients were enrolled between May 23, 2006 and January 31, 2008 in nine rural district hospitals located in the Centre Province and followed-up over 2 years. Eligibility criteria included having a HIV-1 group M infection, being older than 18 years and being eligible for ART, in accordance with national guidelines and WHO 2006 recommendations [13]. Participants were requested to attend clinical visits which included an interview and a physical examination by a healthcare professional at weeks 0 and 2, months 1 and 3, and every 3 months thereafter.

Socio-economic, psychosocial and behavioural data were collected after the clinical visit at baseline (M0, i.e., date of treatment initiation), and at months 6 and 12, using face-to-face questionnaires administered by community health workers trained by the research team. During face-to-face interviews, patients were asked a series of questions including information about sexual activity and adherence to treatment. Patients also received counseling on ART-adherence and prevention of HIV transmission when initiating ART and at each follow-up visit. Condoms were provided for free in the hospitals’ pharmacies.

Clinical data collected for the purpose of the trial, including HIV clinical stages and CD4 cell count (assessed by Facscount equipment), were also used in this analysis.

The study protocol was approved by the National Ethics Committee of Cameroon and the institutional Ethics Committee of the *Institut de Recherche pour le Développement* (France) and all study participants provided written informed consent.

### Definition of Inconsistent Condom Use (ICU)

Use of condoms during the previous three months was assessed separately for the main partner and for casual partners using a four-level scale: never, sometimes, nearly always or always [14]. Inconsistent condom use (ICU) was defined at each follow-up visit as reporting to have “never”, “sometimes” or “nearly always” used condoms with one’s main or casual partner(s) - either HIV negative or of unknown HIV status in the three previous months.

As information about HIV-status (HIV-positive, HIV-negative or unknown) was only available for the main partner, all casual partners were considered to have unknown HIV status.

### Variables

Variables retained for the analyses were classified into the following five categories:

socio-demographic and economic characteristics: gender, age, educational level, professional status, marital status, place of religion in one’s life and perceived social class. They were assessed using a ten-point visual scale, with higher scores denoting a better perceived social situation (higher income, higher educational level and better job) [15].
clinical characteristics: WHO clinical stage and CD4 cell count.
sexual behavior and reproductive life during the previous three months: number of sexual partners including casual partners, frequency of sexual intercourse, type of sexual partner(s) (main only; casual only, main and casual), condom use, having children and the desire for a/another child.
caregiver-patients relationships: feeling comfortable with doctors (very comfortable, quite comfortable, not very comfortable and not at all comfortable), trust in doctors and trust in other healthcare staff using a three-point Likert scale (no trust, little trust and complete trust) and healthcare staff’s readiness to listen, assessed using a six-point visual scale ranging from 1 to 6 with higher values denoting a higher perceived degree of readiness to listen.
psychosocial variables: adherence to ART, satisfaction about information provided by care staff, binge drinking, experience of discrimination and depressive symptoms.

Adherence to ART was assessed using a validated scale [16] including 13 questions. Patients were asked to list, for each antiretroviral drug, the daily number of prescribed pills they had taken during the 4 days prior to the visit. They were also asked if they had “totally” or “partially” taken their prescribed doses of ART or had “interrupted their treatment for more than two consecutive days” during the 4 previous weeks. Those who reported taking 100% of their prescribed doses in the 4 days before the visit and “had not interrupted their treatment” even once for more than two consecutive days during the 4 previous weeks were classified as “adherent” while those who reported taking less than 100% of their prescribed doses in the previous 4-day period or had ‘partially’ taken their prescribed doses or “interrupted their treatment” at least once for more than two consecutive days during the 4 previous weeks were classified as “non- adherent”. Data on adherence reported during clinical visits 28 days before and 7 days after each face-to-face questionnaire visit were also used to increase the sensitivity of the score to detect non-adherence behaviors. Patients who were classified as adherent using the face-to-face questionnaires but whose clinical data indicated that they had taken less than 100% of their prescribed drug doses during the previous 7 days of the clinical visit were classified as non-adherent. The self-reported measure of adherence was validated using a logistic regression model based on the generalized estimating equation, where the outcome was HIV viral load (undetectable vs. detectable) and the explanatory variable was the self-reported measure of adherence (adherent versus non-adherent). The results showed a significant association between non-adherence and undetectable viral load (OR [95% CI]  = 1.47 [1.17–1.84], p = 0.001).

To assess patient satisfaction about information provided by healthcare staff, two variables were used: 1) readiness to listen of healthcare staff and 2) perceived quality of information provided by healthcare staff assessed using a four-point Likert scale (clear information, quite clear information, unclear information and no information at all).

Finally, patients who reported high healthcare staff’s readiness to listen and reported to have received clear or quite clear information were considered as “satisfied about information provided by care staff”.

Binge drinking was defined as the consumption of three large bottles (two liters with 5,2 milliliters of alcohol per 100 ml) and/or six glasses or more of alcoholic beverages on any one occasion [14]. Depressive symptoms were assessed using the Center for Epidemiologic Studies Depression Scale (CES-D) [17], which enables a global depression score to be calculated, ranging from 0 to 60, with higher values denoting more depressive symptoms.

### Statistical Analysis

We first estimated the global proportion of ICU during the first 12 months of ART and the frequency of ICU at each visit. The proportion of ICU over a given period was calculated as the number of visits at which the study population reported ICU over the number of visits performed. Mixed-effect logistic regression models - which enable the correlations between repeated measures to be taken into account - were used to estimate the relationship between adherence and sexual risk behaviors. As the proportion of patients reporting ICU during the first 12 months of ART was not significantly different between the two treatment approaches (i.e., clinical monitoring versus clinical plus laboratory monitoring) when performing a chi-square test (p-value = 0.20), both groups were pooled in analysis. All explanatory variables with a *P*-value <0.20 in the univariate analyses were eligible to enter the initial multivariate model. The final multivariate model was obtained by using a backward stepwise procedure based on the log-likelihood ratio to eliminate non-significant (*P*>0.05) variables from the initial model.

Statistical analyses were performed using SPSS v15.0 (SPSS, Inc., Chicago, Illinois, USA) and Intercooled Stata 10 (StataCorp LP, College Station, Texas, USA) software packages.

## Results

### Characteristics of HIV Patients

All 459 PLWHA who initiated ART in the Stratall ANRS 12110/ESTHER trial agreed to participate in the socio-behavioral study. During the first 12 months of ART, data on sexual behaviors were available for 435 patients (95%). Among them, 308 (71%) were women, median [interquartile range, IQR] age was 36 years (30–45), 101 (23%) reported being married and 213 (49%) patients had an educational level higher than secondary school. Patients who had no data on sexual behaviors (n = 24) were not significantly different compared with those for whom data were available in terms of gender, age and CD4 cell count at ART initiation (data available on request). Among patients having data on sexual behaviors, 241 (55%) reported sexual activity at least once during all follow-up visits while 194 (45%) reported sexual abstinence during the first 12 months of ART. Those patients who reported sexual activity were younger than their sexually abstinent counterparts (median [IQR] = 35 years [30–42] versus 39 years [31–47], P<10^−3^ (test de Mann-Whitney)); they were more frequently married, reported more often the desire to have a/another child, and had a higher median CD4 cell count. No significant differences were however detected concerning gender and WHO clinical stage.

### Study Population

Among the 241 respondents who reported to have been sexually active during the first 12 months of follow up, 29 (12%) declared that they had sex only with HIV positive partner(s). We therefore conducted our analysis on the remaining 212 (88%) patients (i.e., accounting for 344 visits) who reported at least once to have had sexual intercourse during the first 12 months of treatment with their main or casual partner(s) - either HIV-negative or of unknown HIV status. Among these 212 patients, 153 (72%) - accounting for 212 visits (62%) - reported inconsistent condom use.

### Baseline Characteristics of the Study Population (n = 212)

The median age [IQR] of patients was 35 [29–43] and more than a third (69%) were women. A quarter (25%) were married and approximately half (51%) reported having a professional activity. Regarding HIV-related characteristics, a quarter (26%) of patients were at WHO clinical stage IV, the overall median [IQR] CD4 cell count per mm^3^ was 200 [82–393] and the median [IQR] score of depressive symptoms was 22 [15]–[29]. Concerning sexual behaviors and reproductive life characteristics, 53% of patients declared to be sexually active at baseline (31% had only a main partner, 12% had at least one casual partner, 10% had both main and casual partner(s) while 47% had no partner). A quarter of patients who had a partner reported more than one sexual act per week. The majority of patients (81%) had at least one child and 12% desired to have a/another child. Baseline characteristics of the study population, stratified by sex, are presented in Table 1.

**Table 1:** Baseline characteristics of HIV-infected patients reporting sex with main or casual partner(s) - either HIV negative or unknown status during the first year of antiretroviral therapy in Cameroon (n = 212).

	Women (n (%)or median [IQR*])(n = 147)	Men (n (%)or median [IQR*])(n = 65)	p-value
Age (years)	40 [33–45]	33 [28–40]	<10^−3^
Educational level >primary school	76/144 (53)	36/59 (61)	0.28
Professionally inactive	66/135 (49)	20/60 (33)	0.04
Married	24/147 (16)	28/65 (43)	<10^−3^
Having children	109/140 (78)	62/65 (95)	0.001
Perceiving one’s social class as low	52/144 (36)	18/63 (29)	0.29
Sexually active	65/140 (46)	26/60 (43)	0.69
>1 partner	11/140 (8)	13/60 (22)	0.01
Desire to have another child	20/140 (14)	6/64 (9)	0.38
Binge drinking	16/137 (12)	16/60 (27)	0.01
Depressive symptoms	22 [16]–[29]	23 [15]–[28]	0.95

* IQR: interquartile range.

### ICU and Adherence Trends

Of the total 344 visits corresponding to 212 HIV-patients who reported at least once that they had sex during the first 12 months of treatment with main or casual partner(s) - either HIV-negative or of unknown HIV status (study population), 113 (32%), 112 (33%) and 119 (35%) were visits at enrollment (M0), M6 and M12 respectively. During follow-up, ICU was reported in 212 visits (corresponding to 153 patients), broken down as follows: 86/113 visits (i.e. 76% of visits) at M0, 56/112 (50%) at M6 and 70/119 (59%) at M12 respectively. Adherence to ART was reported at 67/112 visits (i.e. 60% of visits) at M6 and at 79/119 (66%) at M12. The proportion of ICU at visits where adherence to ART was reported was 45% (30/67) at M6 and 57% (45/79) at M12 versus 58% (26/45) at M6 and 63% (25/40) at M12 at visits where non-adherence was reported. To test whether the relationship between non-adherence and ICU could change over time, we tested the interaction between time and adherence over the period M6–M12. It was not significant, confirming that this relationship did not indeed change over time.

### Factors Associated with ICU

In univariate analysis (Table 2), several socio-economic and psychosocial characteristics were found to be significantly associated with a higher risk of ICU as follows: being married, perceiving one’s social level as low, being professionally inactive and having depressive symptoms. Conversely, none of the clinical characteristics was significantly associated with ICU. Among caregiver-patient relationship variables, high score for healthcare staff’s readiness to listen, having trust in healthcare staff and being satisfied about information provided by healthcare staff were all significantly associated with ICU. Other sexual and reproductive behavioral characteristics significantly associated with ICU included the desire to have a child, having sex more than once a week, having more than one sexual partner, having sex only with casual partner(s) and having sex with both one’s main and casual partner(s) (compared with one’s main partner only). Finally, both adherence and non-adherence to ART were significantly associated with a reduced risk of ICU compared with baseline (i.e., at ART initiation).

**Table 2:** Factors associated with inconsistent condom use among HIV-infected patients reporting sex with a main or casual partner(s) - either HIV negative or of unknown status during the first year of antiretroviral therapy in Cameroon: univariate and multivariate analyses using mixed-effect logistic models (212 patients, 344 visits).

	Number of visits (%) or median[IQR]	Number of patients	OR [95%CI]	p-value	AOR [95%CI]	p-value
***Socio-demographic and economic characteristics***						
Female gender	240 (70)	147	1.10 [0.52–2.31]	0.81		
Age – OR per 10-year increase	35 [30–43]		1.21 [0.83–1.78]	0.32		
Educational level >primary school*	183 (53)	112	0.62 [0.31–1.23]	0.17		
Professionally inactive*	128(37)	85	2.33 [1.14–4.76]	0.02		
Married *	83 (24)	53	3.04 [1.24–7.45]	0.01		
Perceiving one’s social class as low^c^ *	69 (31)	53	2.59 [1.28–5.23]	0.01	2.15 [1.05–4.30]	0.03
Perceiving the place of religion in one’s life as very important	189 (55)	117	0.67 [0.34–1.34]	0.26		
***HIV-related characteristics***						
WHO clinical stage IV	87 (25)	55	1.22 [0.55–2.67]	0.50		
Baseline CD4 count – OR for a 100 cells/mm3 increase	210 [82–407]		1.03 [0.89–1.20]	0.67		
Baseline CD4 count <200 cells/mm3	167 (49)	107	0.74 [0.38–1.48]	0.88		
***Sexual behaviors and reproductive live characteristics***						
More than one sexual act per week*	50 (15)	45	5.72 [2.03–16.10]	0.004	4.31 [1.36–13.67]	0.02
More than one sexual partner *	51 (15)	45	3.32 [1.29–8.52]	0.01		
Having (a) casual partner(s)a*	91 (26)	74	4.92 [2.28–10.85]	<10^−3^		
Type of sexual partner(s)*						
*- Having main partner only (ref)*	246 (73)	166	1.00		1.00	
*- Having casual partner(s) only*	46 (14)	40	4.18 [1.54–11.35]	0.005	3.73 [1.34–10.35]	0.01
*- Having main and casual partners*	44 (13)	39	5.64 [1.94–16.43]	0.002	4.96 [1.77–13.89]	0.002
Having children	280 (81)	176	1.54 [0.64–3.75]	0.34		
Desire to have a/another child*						
*- Yes*	66 (20)	53	3.30 [1.34–8.14]	0.01	3.25 [1.38–7.64]	0.007
*- No (ref)*	247 (73)	170	1.00		1.00	
*- Unable to procreate*	25 (7)	21	1.39 [0.42–4.65]	0.59	1.56 [0.50–4.87]	0.45
***Caregivers-patients relationship related characteristics***						
Feels very comfortable with doctors	243 (71)	158	0.90 [0.43–1.88]	0.78		
High healthcare staff’s readiness to listen*	219 (64)	150	0.41 [0.19–0.86]	0.02		
High trust in doctors (complete trust)	304 (88)	192	0.74 [0.19–2.91]	0.67		
High trust in health care staff (complete trust)*	294 (85)	188	3.16 [1.04–9.58]	0.04		
***Psychosocial characteristics***						
ART treatment and adherence*						
*- Baseline (treatment not yet initiated) (ref)*	113 (33)	113	1.00		1.00	
*- Treated and non-adherent*	85 (25)	78	0.43 [0.20–0.92]	0.03	0.58 [0.26–1.29]	0.19
*- Treated and adherent*	146 (42)	115	0.30 [0.15–0.58]	<10^−3^	0.38 [0.19–0.76]	0.006
Satisfied about information provided by care staff*	220 (64)	150	0.49 [0.25–0.95]	0.04		
Binge drinking^b^ *	56 (16)	41	2.25 [0.95–5.65]	0.07		
Experience of discrimination*	100 (29)	76	1.73 [0.87–3.45]	0.12		
Depressive symptoms – OR per 1-point increase in the CES-D^d^ score*	15 [9]–[25]		1.08 [1.04–1.12]	<10^−3^		

OR  =  crude odds ratio, AOR  =  adjusted odds ratio, IQR: interquartile range,

* included in multivariate analysis,

a during the previous 12 months,

b consumption of three big bottles and/or six glasses of alcoholic beverages or more on any one occasion,

c level 1 or 2 on a ten-point scale [15],

d score range 0–60, higher values denote more depressive symptoms [17].

After adjustment in multivariate analysis for the frequency of sexual acts, type of sexual partner (i.e., having a main partner only, casual partner(s) only or having both a main and casual partners), perceived social class and desire for a child, adherence to ART was independently associated with a lower risk of ICU compared with baseline (AOR [95% CI] = 0.38 [0.19–0.76]; P = 0.006) (Table 2). In order to test whether satisfaction about the information provided by healthcare staff (which has been found to be related to adherence [18]) was on the same causal path leading to ICU, we entered it in the final model instead of adherence. This variable remained significantly associated with ICU (p<0.03). However, when then adding adherence in the model, this variable was no longer significant. To verify whether any factors could have had a different impact on the outcome because of gender, we tested all interactions between gender and eligible risk factors for ICU but none was significant in the final model.

## Discussion

In this population of Cameroonian HIV-infected adults followed up in nine rural district hospitals, we documented a reduction in ICU during the first year of follow-up after ART initiation. This is consistent with the results of meta-analyses conducted both in high and low-resource settings [7], [8]. The present study also highlights the fact that after adjustment for known correlates of ICU, patients who were adherent after commencing ART were less likely to report ICU than before commencing it. However the increase in ICU observed in ART adherent patients from M6 to M12 (though not significant) is consistent with previous research (outlined below), showing that continued adherence to ART in the long term can lead to increased risk behaviors as a result of improved health and beliefs about reduced infectiousness [19].

In terms of PLWHA sexual behaviors, the impact of ART initiation has been studied in several longitudinal studies in Sub-Saharan Africa settings. A decline in the proportion of sexual risk behaviors 6 months after ART initiation has been highlighted in Uganda and South Africa [9], [20]. Similar results were also observed in Kenya after 12 months of treatment [21] and in South Africa after 8 years [9]. Among all the available studies, only a longitudinal study carried out by Diabate et al. in the Côte d’Ivoire showed an increase in the frequency of sexual risk behaviors among PLWHA after six months of follow-up [22]. Few longitudinal studies have however examined the relationships between initiating ART, ART adherence and sexual risk behaviors among HIV-infected Sub-Saharan African adults. In the present study, we distinguished two sub-groups among patients after ART initiation: those who were “adherent” to ART and those who were “non-adherent”. Our study suggests that, after multiple adjustment, ART-adherent patients were less likely to report sexual risk behaviors while non-adherent patients had a similar risk to that reported at baseline. This may suggest that patients who were adherent to ART were also “adherent” to HIV prevention messages provided during counseling sessions [6]. Sustained adherence to ART is considered to be one major condition for Treatment as Prevention (TasP) strategy [4] because it makes it possible to both control HIV viral load and reduce the likelihood of HIV transmission through sexual intercourse. However, the fact that satisfaction about the information provided by healthcare staff was associated with ICU when removing the adherence variable from the final multivariate model - but not when both were present in the model - confirms two things: first, that dissatisfaction with the information provided by healthcare staff is a predictor of ICU and second, that it constitutes an important component of adherence behaviors [18]. As adherence behaviors are not only determined by the quality of care delivered but also by factors which are external to the healthcare system, like stigma and disclosure [6], [23], we can assume that those factors probably also affect sexual behaviors.

Furthermore, the study’s findings show that participants who desired to have children were more likely to report inconsistent condom use [9], [24]. These results are illustrated by the increase of the proportion of patients who desired to have children from 14% at M0 to 25% and 20% at M6 and M12 respectively. This suggests that ART has a positive effect on the desire for children among ART-treated PLWHA [25] but may therefore encourage unprotected sex. In addition, motherhood is such a fundamental social value in Africa that women’s desire for children may be stronger than the fear of mother-to-child HIV transmission [26]. Our results may also be considered in parallel with those from a study recently conducted in Sub-Saharan Africa which showed a higher rate of pregnancy among couples with at least one partner receiving ART compared with couples with no ART [27].

As previously found in other studies, the frequency of sexual intercourse per week [28], [29], the perceived social class as well as the type of sexual partner [14] were also found to be associated with unsafe sex in our study.

Despite these interesting results, some study limitations need to be recognized. Sexual behaviors and adherence assessment are based on self-reports which are known to be affected by social desirability bias [20]. While this bias was better minimized in terms of adherence by using a specific algorithm based on several questions, this was not the case for inconsistent condom use. Bias induced by face to face interviews was however reduced by training counselors in the use of non-judgmental approaches. Another limitation of the study is that we could not distinguish whether or not sexual behaviors were different according to the type of casual partners (e.g. sex-trade partners versus other partners). Indeed, it is possible that condom use with sex-trade partners was reported very differently than for any other type of partnership. Despite this weakness, the proportion of reported ICU was similar to that reported in other studies in Cameroon [14]. Finally, as this study was part of a clinical trial focusing in which patients who were unlikely to attend clinical visits were not eligible might have reduced the relationship strength between adherence to treatment and ICU. As in any trial, a problem of external validity may be present, affecting the generalization of these results in the operational research context. Hence it is possible that the proportion of ART-adherent PLWHA may have been higher than that which would have been obtained in an operational context.

In conclusion, this study highlights that adherence to ART is associated with a lower risk of ICU but this result needs to be interpreted carefully. As adherence behaviors are not only determined by problems with the healthcare systems but also by social barriers encountered by patients in their daily life, counseling should not only be ART adherence-centered but patient-centered, including sexual risk minimization and psychosocial support.
