# Correction: Adherence as a Predictor of Sexual Behaviors in People Living with HIV/AIDS during the First Year of Antiretroviral Therapy in Rural Cameroon: Data from Stratall ANRS 12110/ESTHER Trial

**DOI:** 10.1371/journal.pone.0323693

**Published:** 2025-05-08

**Authors:** Gilbert Ndziessi, Sylvie Boyer, Charles Kouanfack, Julien Cohen, Fabienne Marcellin, Jean-Paul Moatti, Eric Delaporte, Bruno Spire, Christian Laurent, Maria Patrizia Carrieri, for the Stratall ANRS 12110/ESTHER Study Group

The name of a member of the Stratall ANRS 12–110/ESTHER Study Group was incorrectly listed as J. Fokom. The correct name is Joël Fokom Domgue.

The corrected Acknowledgments should read: The authors would like to thank the participating hospitals and their medical teams for their strong involvement in the project, as well as all participating patients. Thanks also to Jude Sweeney for the English revision and editing of the manuscript.

The Stratall ANRS 12110/ESTHER Study Group:

M. Biwolé-Sida, C. Kouanfack, S. Koulla-Shiro (Central hospital, Yaoundé, Cameroon); A. Bourgeois, E. Delaporte, C. Laurent, M. Peeters (IRD, University Montpellier 1, UMI 233, Montpellier, France); G. Laborde-Balen (French Ministry of Foreign Affairs, Yaoundé, Cameroon); M. Dontsop, S. Kazé, J-M. Mben (IRD, Yaoundé, Cameroon); A. Aghokeng, M.G. Edoul, E. Mpoudi-Ngolé, M. Tongo (Virology Laboratory, IMPM/CREMER/IRD-UMI 233, Yaoundé, Cameroon); S. Boyer, M.P. Carrieri, F. Marcellin, J-P. Moatti, B. Spire (INSERM, IRD, University Marseille, UMR 912, Marseille, France); C. Abé, S-C. Abega, C-R. Bonono, H. Mimcheu, S. Ngo Yebga, C. Paul Bile (IRSA, Catholic University of Central Africa, Yaoundé, Cameroon); S. Abada, T. Abanda, J. Baga, P. Bilobi Fouda, P. Etong Mve, G. Fetse Tama, H. Kemo, A. Ongodo, V. Tadewa, HD. Voundi (District Hospital, Ayos, Cameroon); A. Ambani, M. Atangana, J-C. Biaback, M. Kennedy, H. Kibedou, F. Kounga, M. Maguip Abanda, E. Mamang, A. Mikone, S. Tang, E. Tchuangue, S. Tchuenko, D. Yakan (District Hospital, Bafia, Cameroon); J. Assandje, S. Ebana, D. Ebo’o, D. Etoundi, G. Ngama, P. Mbarga Ango, J. Mbezele, G. Mbong, C. Moung, N. Ekotto, G. Nguemba Balla, G. Ottou, M. Tigougmo (District Hospital, Mbalmayo, Cameroon); R. Beyala, B. Ebene, C. Effemba, F. Eyebe, M-M. Hadjaratou, T. Mbarga, M. Metou, M. Ndam, B. Ngoa, EB. Ngock, N. Obam (District hospital, Mfou, Cameroon); A. M. Abomo, G. Angoula, E. Ekassi, Essama, J.J. Lentchou, I. Mvilongo, J. Ngapou, F. Ntokombo, V. Ondoua, R. Palawo, S. Sebe, E. Sinou, D. Wankam, I. Zobo (District hospital, Monatélé, Cameroon); B. Akono, A. L. Ambani, L. Bilock, R. Bilo’o, J. Boombhi, F.X. Fouda, M. Guitonga, R. Mad’aa, D.R. Metou’ou, S. Mgbih, A. Noah, M. Tadena, Ntcham (District hospital, Nanga Eboko, Cameroon); G. Ambassa Elime, A.A. Bonongnaba, E. Foaleng, R.M. Heles, R. Messina, O. Nana Ndankou, S.A. Ngono, D. Ngono Menounga, S.S. Sil, L. Tchouamou, B. Zambou (District hospital, Ndikinimeki, Cameroon); R. Abomo, J. Ambomo, C. Beyomo, P. Eloundou, C. Ewole, Joël Fokom Domgue, M. Mvoto, M. Ngadena, R. Nyolo, C. Onana, A. Oyie (District hospital, Obala, Cameroon); P. Antyimi, S. Bella Mbatonga, M. Bikomo, Y. Molo Bodo, S. Ndi Ntang, P. Ndoudoumou, L. Ndzomo, S.O. Ngolo, M. Nkengue, Nkoa, Y. Tchinda (District hospital, Sa’a, Cameroon).
